# The asthma epidemic and our artificial habitats

**DOI:** 10.1186/1471-2466-5-5

**Published:** 2005-03-31

**Authors:** Wasim Maziak

**Affiliations:** 1 Syrian Center for Tobacco Studies, Aleppo, Syria; 2 Institute for Epidemiology and Social Medicine, University of Muenster, Germany

## Abstract

**Background:**

The recent increase in childhood asthma has been a puzzling one. Recent views focus on the role of infection in the education of the immune system of young children. However, this so called hygiene hypothesis fails to answer some important questions about the current trends in asthma or to account for environmental influences that bear little relation to infection.

**Discussion:**

The multi-factorial nature of asthma, reflecting the different ways we tend to interact with our environment, mandates that we look at the asthma epidemic from a broader perspective. Seemingly modern affluent lifestyles are placing us increasingly in static, artificial, microenvironments very different from the conditions prevailed for most part of our evolution and shaped our organisms. Changes that occurred during the second half of the 20th century in industrialized nations with the spread of central heating/conditioning, building insulation, hygiene, TV/PC/games, manufactured food, indoor entertainment, cars, medical care, and sedentary lifestyles all seem to be depriving our children from the essential inputs needed to develop normal airway function (resistance). Asthma according to this view is a manifestation of our respiratory maladaptation to modern lifestyles, or in other words to our increasingly artificial habitats. The basis of the artificial habitat notion may lie in reduced exposure of innate immunity to a variety of environmental stimuli, infectious and non-infectious, leading to reduced formulation of regulatory cells/cytokines as well as inscribed regulatory pathways. This could contribute to a faulty checking mechanism of non-functional Th2 (and likely Th1) responses, resulting in asthma and other immuno-dysregulation disorders.

**Summary:**

In this piece I discuss the artificial habitat concept, its correspondence with epidemiological data of asthma and allergy, and provide possible immunological underpinning for it from an evolutionary perspective of health and disease.

## Background

Asthma is a major health problem that has reached alarming proportions in the past two decades in western societies [1]. What lies behind the recent increase in asthma in affluent societies is still an area of lively debate, but its rapid changing patterns and huge variation across populations favor environmental explanations [1-3]. While assessment of different old and new exposures is continuing, a unifying paradigm remains elusive, so as a guiding principle for prevention. The simultaneous increase of all forms of allergic disease on the other hand, argues for a change in host susceptibility/resistance [4].

The apparent association between asthma and western lifestyle has lead to numerous studies trying to link novel or increased exposures associated with westernization-modernization, especially those occurring during childhood, to asthma. For example, exposure to gas cooking, tobacco smoke, trans fatty acids, domestic animals, and allergens were found to influence respiratory health, but did not provide a conclusive answer to the current trends in asthma [5-10]. While it is early to discard the role of these and other factors, a major contribution of asthma research during the past two decades lies in the elucidation of its heterogeneous and multi-factorial nature, where different exposures have different roles and relevance depending on the target population, setting, and disease course.

## Discussion

### 1- The hygiene hypothesis and asthma

Since its introduction in the nineties, the hygiene hypothesis (HH) continues to generate enthusiasm among asthma researchers as the most comprehensive theoretical framework, by which the relation between suspected environmental factors and allergy can be tested [11-13]. This hypothesis originated from the coupling of observations on the allergy-protective effect of sibship size/birth-order with the emerging concept of helper T cell polarization into two counter-regulatory subsets; pro-infection Th1, and pro-allergy Th2 [14,15]. Backed by some experimental and clinical evidence [16-18], the hygiene hypothesis suggests that the recent rise in allergic disease among children in affluent societies is due the preferential programming of the T cell repertoire towards pro-allergy Th2 responses, brought by the decline in infections (increased hygiene, immunization, decreased sibship size, antibiotic use) [13-15]. With the increasing recognition of the role of T regulatory cells (Tregs) and cytokines in the pathogenesis of allergic inflammation, the hygiene paradigm has been extended recently integrating infection's role in generating such cells and mediators [19-21].

Asthma trends however, did not fit well with the hygiene model, which failed to explain the urban predominance of asthma, the increase in non-allergic asthma, the disparity between atopy and allergy in some populations, and the asthma inducing properties of some infections [22-27]. Specifically, studies looking at the infection-asthma relationship failed to yield a consistent pattern so far [28-34], prompting David Strachan, the father of the hygiene hypothesis, to conclude that "the totality of current evidence from cross sectional and longitudinal studies of common specific and non-specific infectious illnesses in infancy and childhood offers no support for the hygiene hypothesis" [11]. Other studies doubted even the fundamentals of the HH showing that the effect of siblings is not universal, or could have been programmed in utero rather than being a marker of childhood infection [35,36]. Perhaps the hygiene model's major shortcoming lies in its concentration on only one aspect (infection) of the drastic change that touched upon every detail of life in western societies in the past few decades. It also adopted a mechanistic approach for the study of adaptive immune responses and the relation between exposure-outcome, where an array of potential interactions are reduced to a single level; Th1-Th2 counter-regulation, siblings-infection, daycare attendance-infection, dog ownership-endotoxin, farming-endotoxin, etc. In brief, the search for a holy grail in the asthma epidemic may need to be replaced by the conceptualization of a more generalizing notion that allows for the consideration of multitude of factors within an ever changing environment.

### 2- Asthma and our artificial habitats

The hygiene model on the other hand, involved an evolutionary logic alerting us to the negative potential of sudden elimination of exposures that have shaped throughout the ages our organs and systems [37]. Health from such perspective is not about abstract assessment of the relation between exposure and outcome, or eliminating harmful exposures, but about seeing the whole dynamics of our interaction with our novel habitats [38]. Because of the slowness of adaptive evolutionary machinery, of particular interest according to this perspective are lifestyle factors that either witnessed a rapid change in recent times or represent an obvious departure from the conditions prevailed for most part of our evolution, i.e. factors likely to exemplify the discordance between our "Stone Age" genes and "Space Age" living conditions [37,38].

General trends of asthma show that populations who conserved elements of ancient lifestyles have low levels of asthma. The north-south, urban rural, gradients in asthma occurrence have been extensively documented [24,37,39-43]. In additions, studies in Africa show that some populations seem to be protected from asthma regardless of atopic predisposition or parasitic infection (both are Th2-related) [20,22,44-46]. This indicates that some environmental influences associated with more traditional lifestyles are conferring respiratory resistance to stimuli that could have lead otherwise to clinically relevant airway inflammation. Within western societies furthermore, lower levels of asthma were found in families with traditional lifestyles [47,48] and higher levels of asthma were found among obese or less physically fit children and adults [49-51]. Because not all these observations can be explained by variability in infection, or any other single factor for that matter, a broader concept seems more plausible.

Modern life is increasingly placing us in static, artificial, micro-niches optimized for our convenience, but which bear little resemblance to the dynamic inputs provided by the environments that nurtured our evolution. In other words, we are increasingly living within an array of artificial habitats designed to handle us very well, but we may well not be equipped to handle them. Asthma according to this view, becomes a manifestation of our respiratory maladaptation to modern lifestyles. Changes that occurred during the second half of the 20^th ^century in industrialized countries with the spread of central heating/conditioning, building insulation, hygiene, TV/PC/games, manufactured food, indoor entertainment, cars, medical care, and sedentary lifestyles all seem to be depriving our children from the essential inputs needed to develop normal airway function (resistance).

### 3- Epidemiology of asthma from a new perspective

The suggested view -called here the artificial habitat (AH)- can provide alternative interpretations to existing data starting with the protective effect of sibship size/birth-order, which is one of the landmark observations of the HH that has been ascribed to increased exposure to infection [52]. It can be postulated that the mechanism of protection of siblings (especially older males) is related to their importance to the child's level of physical activity as well as ability to spend more time outdoors (i.e. in a more dynamic environment). This is particularly relevant to children living in dangerous neighborhoods, such as inner cities in the US, where the spread of asthma represents one of the main challenges to the hygiene paradigm [53]. Indeed, Andrew-Aligne and colleagues found that the higher prevalence of asthma among inner city black children is not due to race or low income per se, but to their living in an urban setting [54]. Additional intriguing support to the AH notion comes from the two largest studies looking at the effect siblings on the occurrence of allergy, whereby a stronger protective effect was observed for brothers than for sisters [55,56]. While exposure to infection cannot be expected to be related to sibling's gender, activity and outdoor time may well be influenced by this factor. By the same token, birth order can determine, among other things (e.g. social development, healthy food availability), the child's level of activity (how many playmates the child have) and ability to spent time outdoors. As new evidence are emerging against the HH's assumption considering the infection-related effect of the sibship size/birth-order, by showing for example that the role of birth order is independent of sibship size [57], and that in the same population sibship size can protect against asthma while infection predispose to it [31,58], the AH concept seems to offer an alternative explanation.

Another important observation of the hygiene paradigm concerns the protective effect of early daycare attendance on later development of asthma, which is ascribed to increased exposure to infection [28]. Alternatively, it can be argued that the daily routine at a daycare center would be different in many aspects from home, in addition to exposure to infection. Reducing potential differences in activity, exposure, socio-behavioral development, and parental attitudes between those who do and don't attend daycare to mere infection seems over-simplistic. The AH concept looks at this observation from a broader angle involving a mixture of lifestyle and developmental factors. Related to this issue is the argued window of opportunity in early infancy for the protective effect of daycare attendance/infection [59,60], which is connected to a critical period of immune education [13]. By its own nature in contrast, the AH view is consistent with the notion that environmental signals throughout the lifespan can affect the risk as well as the course of asthma and allergy.

On a different juncture of asthma research, a multitude of recently published reports show lower rates of asthma and atopy in children raised on a farm [61-68]. Heavily influenced by the HH, these observations were largely attributed to increased exposure to bacterial components found in barns or farm milk (endotoxin in particular) [67-69], forgoing that children raised on a farm have very different lifestyles from children growing in inner cities in the US for example, where infection is also commonplace [70]. For example, one of the landmark farming studies has shown that endotoxin levels in children's mattresses were inversely associated with the occurrence of hay fever and atopic asthma [67]. However, leukocytes of children exposed to high levels of endotoxin produces less Th2 suppressing cytokines (mainly interleukin 10), arguing against the endotoxin-hygiene paradigm [67,71,72]. From the AH perspective however, a farm is the closest we can get in today's' western societies to conditions prevailed for most part of our evolution. Such an environment can provide ample opportunities of behaviors and exposures different from those of modern urban life.

The AH perspective is consistent with the assumption that time spent outdoors and level of physical activity should be protective against the development of asthma. A recent study by McConnell and colleagues however, has shown just the opposite, where the risk of developing asthma was positively associated with number of sports played and time spent outdoors [73] However, when participating communities were separated according to their level of atmospheric ozone, this association was only seen in the high ozone levels communities, while number of sports played and time spent outdoors seem to be protective in the low ozone communities [73]. On the other hand, twines in the Odense study who participated in conditioning exercise had a decreased risk of asthma compared to the more sedentary co-twins [51]. The physiological underpinning of the effect of activity on asthma can be partly elucidated by the work of Feldberg and colleagues and Togias and colleagues, who showed that disruption of dynamic breathing (static breathing without deep breaths or sighing) can lead to bronchial hyper-responsiveness (BHR), the pathophysiological hallmark of asthma [74,75]. Furthermore, recent evidence shows that obesity and weight gain are associated with increased risk of BHR [76], providing more insight on possible ways by which sedentary life factors can co-interact to predispose to asthma.

Finally, the AH concept can offer an explanation for some puzzling observations, such as the protective effect of dog ownership on asthma [77,78]. While the HH proponents looked for explanation in endotoxin levels associated with dog ownership, but with conflicting results so far [79], one can argue that the change in lifestyle (of children particularly) associated with dog ownership can be responsible (more playing, more time out, emotional interaction, as well as exposure to dog's constituents). Generally, families who opt to have a dog may be different from those who don't in being more active and outgoing. The focus here on activity and outdoor time is because these factors are clearly envisioned. Other aspects of traditional lifestyles may be just as important, such as household air exchange (e.g. affecting allergens, pollutants, humidity), nutritional habits (e.g. breast feeding), as well behavioral adaptations.

### 4- An innate control of asthma and allergy

Now how can the AH view be related to what we know about the immunopathology of asthma and allergy? Asthma is an immunological disorder with a predominant Th2 inflammatory response in the airways. This Th2 response is thought to be a remnant of our ability to expel parasites abundant in the cradle of human evolution, the tropical savannah [80]. Indeed, evidence exist showing that Th2 pro-inflammatory genetic alleles are more prevalent in populations with a tropical origin than those with a temperate one [81]. It is possible that the need to deal with a wide variety of pathogens may have meant that the activation threshold of Th2 responses has to be set low, leading to many false alarms to non-pathogenic particles and giving rise to asthma. Evolutionary logic indicates however, that a trait with a potential to endanger air passage into our vital respiratory organs would not have been selected, had some regulatory mechanisms not been in place. Studies on the initial phase of allergic sensitization show that a transient low-level IgE (the atopic antibody) response to inhalant antigen occurs in normal children, with those who do not develop allergy down-regulate it in the first years of life [82-84]. Asthma in this regard, becomes a manifestation of breakdown of regulatory mechanisms at respiratory mucosal surfaces.

But let's take one step back to look at another recent puzzling trend; the increase of Th1 autoimmune disorders such as type-1 diabetes and multiple sclerosis in western societies [21]. Recent evidence suggests that that the two groups (Th1 and Th2 mediated diseases) can be associated in individuals [85-88], arguing against the Th1-Th2 counter-suppression of the HH, and favoring a common ground of faulty regulation. Such developments were picked up by proponents of the HH to suggest that hygiene can work through depriving the immune system from signals necessary for the development of regulatory pathways/cells capable of dampening both Th1 and Th2 responses [89,90]. While this can be true, the focus on infection yet again is a reductionistic view likely to suffer the same shortcomings of the original Th1-Th2 counter-regulation of the HH. At the same time, advances made in immunology were unraveling the central role of the innate immune system in orchestrating immune responses [91]. In particular, antigen presenting cells, such as dentritic cells (DCs), can engage infectious components with their Toll-like receptors (TLRs) (a group of ancient immune recognition molecules) leading to activation of adaptive immune responses and induction of regulatory cells and mediators [92,93]. In their turn, T regulatory cells (Tregs), which are induced naturally or by elements of innate immunity are able to regulate all types of adaptive immune responses as well as influence DCs activation and regulation [94,95]. Interestingly, it looks that none of the Th1, Th2, or Treg-inducing functions of DCs is an intrinsic attribute that is not sensitive to instructions from the surrounding environment [96,97]. Without getting into the details of this fascinating and still unfolding field, the move from the see/saw mechanistic counter-regulation of adaptive Th1-Th2 responses to elements of innate immunity offers an evolutionary sound and possibly robust checking mechanism (break) against inappropriate responses (e.g. Th2 responses to non-pathogenic elements) at our vital airways. The ability of DCs to be activated in response to danger signals induced by stress, damage, or necrotic cell death [98], and the role of DCs at the gastrointestinal tract in the development of mucosal tolerance [99], broadens their possible range of involvement with different environmental stimuli and thus their contribution to immune homeostasis at the respiratory surface. For example, heat-shock proteins (hsps, which are highly conserved cellular proteins that can be produced by thermal stimuli, physical activity, or other stresses) can activate DCs as well as contribute to T cell regulation of inflammatory responses [100,101].

Taken together, it can be suggested according to the AH concept that dynamic/traditional lifestyles with associated exposures can ensure constant challenge of DCs and other elements of innate immunity giving rise to immune responses, but at the same time maintaining adequate turnover of regulatory cells and cytokines and inscribed regulatory pathways. This ongoing activation of regulatory pathways can help maintain healthy control of non-functional Th2 responses at the respiratory surface. The DC-orchestrated dynamic balance between Th2 responses and regulatory mechanisms is likely to influence all phases (initiation, effector) of inflammation in the airways, and throughout the lifespan of the individual.

## Summary

While it offers no specific explanation to different asthma trends and variations, the suggested AH notion provides a generalizing scheme for the study of asthma, and provides novel insights for existing epidemiological observations. According to this perspective there is no single answer to the asthma epidemic, but different factors have different relevance depending on the population and environment in focus. In addition to being free from the HH one-dimensional approach for the relation between exposure-outcome, this view is evolutionary-driven allowing to place the asthma epidemic within the wider perspective of increasing discordance between us and our dramatically changing environments. Sedentary lifestyles, static indoor microenvironments, and automation of the food chain are apparently not only predisposing us to obesity ad cardiovascular disease but also depriving our respiratory system from many stimuli necessary for the development of normal airway resistance. The immunological basis of the AH notion can lie in the centrality of innate immunity and its ability to respond to different types of environmental stimuli, insuring adequate turnover of regulatory cells and mediators. The evolutionary tenet "the more we change the world the more we stay the same" probably lacks accuracy. Newer environments, constantly confront us with new adaptive challenges that should be looked upon, as in the case of asthma, within the evolutionary context of health and disease.

## Abbreviations

HH- hygiene hypothesis

AH- artificial habitat

DCs- dentritic cells

BHR- bronchial hyper-responsiveness

IL10- interleukin 10

hsps- heat shock proteins

Th1- T helper cell type 1

Th2- T helper cell type 2

Tregs- regulatory T cells

IgE- immunoglobuline E

TLRs- Toll-like receptors

## Competing interests

The author(s) declare that they have no competing interests.

## Authors' contributions

Dr. Wasim Maziak is the sole author and contributor to this manuscript.

## Pre-publication history

The pre-publication history for this paper can be accessed here:


